# Citrate of Soda and the Feeding of Infants

**Published:** 1905-05-20

**Authors:** 


### CITRATE OF SODA AND THE FEEDING OF INFANTS.

Though the artificial feeding of infants has been elevated into a fine art, and though it has been established that by careful additions and dilutions, cow's milk can be very nearly approximated in composition to human milk, such exactitude is too often beyond the reach of poor households. For this reason a knowledge of the value of citrate of soda in reducing the consistency of the curd of cow's milk deserves the widest possible currency. The use of the drug in this connection is by no means new, but it has recently been brought to notice by Dr. Roger[1] who speaks in the highest terms of its beneficial effect in preventing the vomiting of bottle-fed sucklings. The credit for the introduction of the method belongs to Dr. A. E. Wright,[2] and an interesting lecture on the subject was subsequently given by Dr. Poynton.[3]

Briefly stated the fault of cow's milk as compared with that of woman is an excess of proteid and a deficiency in fat and milk-sugar. Analyses differ in detail, but the general fact is established that while human milk contains approximately the same percentage of fat as good cow's milk, the percentage of sugar in it is almost double, and the percentage of proteid about half, that of the latter. The most recent analyses give the following results:

| | Human milk. | | Cow's milk. |
|---|---|---|---|
| Fat ............ | 4 per cent. | ... | 4 per cent. |
| Sugar ......... | 7 per cent. | ... | 4·5 per cent. |
| Proteids ...... | 1·5 per cent. | ... | 3·5 per cent. |

It is clear, therefore, that any dilution which reduces the proteids to their proper proportion must seriously diminish the already deficient sugar and fat-content of the specimen of cow's milk. Nor is this the only difficulty to be met. It is well known that the casein of human milk when curdled by the gastric juices forms a coagulum much more flocculent and of slighter consistency than that derived from cow's milk, and it is this dense curd which is one of the prime obstacles to hand feeding. By the addition of citrate of soda in the proportion of one grain to the ounce of cow's milk the resulting curd is rendered much more flocculent and easy of digestion, and its value may now be considered as substantiated. The drug is cheap—Dr. Poynton gives the price as twopence the ounce—it is harmless, and not easily abused by the most ignorant and careless of mothers. By adopting this method of feeding it is possible to give infants cow's milk in less dilute forms than is otherwise possible, and the danger of underfeeding attaching to large dilutions is avoided. Dr. Poynton observes that the drug when dispensed in watery solution should have added to it enough chloroform water to keep it sterile, otherwise fungi will grow readily on the surface of the solution.

[1] Gazette des Hopitaux, May 2, 1905. [2] Lancet, July 22, 1903. [3] Ibid., Aug. 13, 1904. [4] Diseases of Infancy and Childhood, 1905.

